# Evaluation of the Effectiveness of Close-Knit Medical Alliances on the Integration of HIV Prevention and Treatment in County-Level Areas in China: Protocol for a Delphi Study

**DOI:** 10.2196/76420

**Published:** 2025-11-28

**Authors:** Sixian Du, Yaqing Liu, Qilian Luo, Rongcai Dai, Jiayan He, Yong Yang, Yiqing Yang

**Affiliations:** 1 School of Medicine and Health Management, Tongji Medical College, Huazhong University of Science and Technology, Wuhan, China; 2 Infectious Diseases Department, Linxiang District People's Hospital, No. 116, Nantang Street, Lincang, 677099, China, +86 13987016269; 3 Department of Cardiology, Tongji Medical College, Huazhong University of Science and Technology, Wuhan, China

**Keywords:** HIV, AIDS, integrated health care, Delphi method, public health evaluation, county-level medical services

## Abstract

**Background:**

Integrated care for patients living with HIV in rural and economically disadvantaged regions remains a critical challenge. This study aims to develop a 3D evaluation index system to assess the effectiveness of integrated medical care and prevention for patients living with HIV in county-level regions of China. The framework encompasses disease prevention, clinical treatment, and the integration of medical services with public health interventions.

**Objective:**

This study aims to develop a scientifically validated, 3D evaluation index system for assessing the effectiveness of integrated medical care and prevention for patients living with HIV in county-level regions of China.

**Methods:**

A structured Delphi method will be used to establish consensus among experts on key evaluation indicators. The study will begin with focus group discussions guided by the Valentijn Rainbow Model, followed by multiple rounds of Delphi surveys to refine the indicator system. A total of 75 experts will include clinical professionals, public health specialists, health policy researchers, and representatives from nongovernmental organizations. Experts will participate in iterative rounds, ranking and validating indicators using statistical methods such as the analytic hierarchy process and Kendall W coefficient to assess consensus.

**Results:**

The Delphi study was initiated in April 2025, with 3 rounds of questionnaires distributed between May and September 2025. Data analysis is ongoing, and the finalized county-level HIV integration index system is expected to be published by spring 2026. The study is projected to be completed by January 2026.

**Conclusions:**

The developed evaluation system will offer a comprehensive and scientifically validated tool for assessing health care outcomes in resource-constrained settings, contributing to policy improvements for HIV service integration in China and other low-resource settings globally.

## Introduction

HIV remains a significant global public health challenge, with the integration of medical and preventive services being crucial for effective disease management. By 2021, the global number of people living with HIV had reached 40 million (38‐42.4 million), an increase from 29.5 million (28.1‐31 million) in 2010 [1]. By the end of 2022, an estimated 39 million (33.1‐45.7 million) individuals were living with HIV, two-thirds of whom (25.6 million) resided in the World Health Organization (WHO) African region. Despite ongoing efforts, achieving the global targets of reducing HIV incidence and mortality by 2030 remains highly challenging. Over 40 million people will continue to require lifelong antiretroviral therapy (ART) in the coming decades [2]. Moreover, funding for HIV prevention and control in low- and middle-income countries has declined to US $20.8 billion in 2022—returning to 2013 levels and falling significantly short of the US $29.3 billion needed by 2025 [1]. Since no cure for HIV exists, governments and health care institutions must urgently implement integrated strategies for HIV prevention, diagnosis, treatment, and long-term care. This approach is critical for transforming HIV into a manageable chronic condition, allowing people living with HIV to lead long and healthy lives.

China faces considerable challenges in HIV prevention and control, particularly in integrating medical and preventive services in rural and county-level areas. The 2013 WHO Consolidated Guidelines on the Use of Antiretroviral Drugs for Treating and Preventing HIV Infection emphasized not only the importance of HIV testing and ART coverage but also patient satisfaction, service quality, and the continuity of care between prevention and treatment. The United States was one of the first countries to implement integrated HIV medical and preventive services, with the Office of National AIDS Policy leading the nationwide efforts. Various government agencies, including the Centers for Disease Control and Prevention (CDC), the National Institutes of Health (NIH), and the Health Resources and Services Administration (HRSA), have worked collaboratively to integrate HIV prevention and treatment into broader health care systems [3]. Similarly, sub-Saharan Africa has focused on incorporating HIV services into primary health care frameworks, particularly in managing chronic conditions alongside HIV care [4].

However, China continues to face significant gaps in HIV prevention, treatment, and regulatory integration, with complex social determinants driving the epidemic. HIV prevalence remains high among men who have sex with men, while heterosexual transmission is increasing, often in hidden and hard-to-prevent patterns. Rural areas represent a critical weak link in China’s HIV response due to persistent poverty, inadequate financial support, limited access to high-quality health care and stable housing, fragmented health systems that hinder comprehensive HIV care, poor transportation infrastructure restricting medical access, and low public awareness of HIV prevention, leading to delayed diagnosis and treatment [5]. In rural Guangxi, a significant loss to follow-up has been observed between HIV diagnosis and ART initiation, while studies in parts of Yunnan Province indicate that people living with HIV aged 50 and older often have low health awareness, contributing to continued high-risk behaviors. Addressing these gaps requires a more integrated and comprehensive approach to HIV prevention and treatment [6].

In recent years, the Chinese government has prioritized the development of integrated HIV medical and preventive services. The Healthy China 2030 blueprint, released in 2016, reinforced measures such as expanded HIV testing, ART access, follow-up management, nucleic acid testing for blood safety, and the prevention of mother-to-child transmission, helping to maintain a low overall epidemic level. The National Action Plan on HIV Prevention and Control (2024‐2030) further highlights key priorities, including government leadership, multisectoral collaboration, social mobilization, and public participation. It emphasizes a prevention-first approach while integrating treatment and control, with a focus on high-burden regions and vulnerable populations. The plan also calls for the innovation of medical-preventive integration mechanisms to improve the quality and effectiveness of HIV prevention and control. A key step in achieving these goals is the development of an evaluation framework for HIV medical-preventive integration at the county level. This framework would allow for a more precise assessment and monitoring of HIV prevention, treatment, and regulatory measures, particularly in rural areas where health care resources are limited.

The integration of medical and preventive services is essential for improving the long-term quality of life of people living with HIV, especially in resource-limited settings. However, a scientifically robust evaluation framework for this integration has yet to be developed. Current research on HIV policies has primarily focused on HIV testing rates and ART coverage, often neglecting critical aspects such as patient satisfaction, service coordination, and health education. Future studies should aim to construct a comprehensive evaluation system that encompasses not only traditional indicators (eg, HIV testing rates and ART coverage) [7-9] but also broader dimensions such as health care service coordination, patient experience, and long-term health outcomes. Such a framework would provide policymakers with more holistic evidence for decision-making, ultimately leading to more effective HIV control and long-term disease management [10].

One potential approach is the application of the Valentijn Rainbow Model, which offers a multidimensional perspective on integrated care [11]. The primary objective of this study is to develop a 3D evaluation index system through the Delphi method, based on the Rainbow Model, specifically designed to assess the effectiveness of integrated medical care and prevention for patients living with HIV in county-level regions of China. This comprehensive framework encompasses 3 critical dimensions: disease prevention, clinical treatment, and the integration of medical services with public health interventions. The resulting system aims to provide a scientifically validated measurement tool that can be adapted for evaluating HIV health care outcomes in rural and economically disadvantaged areas globally, thereby offering valuable references for strengthening the global HIV response in resource-constrained settings.

## Methods

### Focus Group Discussions

This study develops a preliminary indicator system framework for the county-level integration of HIV medical and preventive services, guided by the Valentijn Rainbow Model and refined through focus group discussions (FGDs) [12]. The Rainbow Model provides a comprehensive theoretical foundation, emphasizing person-centered and population-centered integration. It comprises 6 dimensions: system integration (macrolevel), organizational and professional integration (mesolevel), clinical integration (microlevel), and normative and functional integration, which facilitate coordination across all levels [13]. This model aligns well with the practical need for seamless medical-preventive service integration in HIV care, ensuring a holistic and patient-centric approach.

The Rainbow Model provides a structured perspective on integrated care, emphasizing that successful service integration requires multilevel coordination. System integration ensures policy alignment and resource allocation at the macro level; organizational and professional integration at the meso level enhances interdisciplinary collaboration and institutional efficiency, while clinical integration at the micro level focuses on seamless patient care. Additionally, normative integration (shared values and goals) and functional integration (harmonized workflows and IT systems) serve as critical connecting mechanisms across levels. By adopting this model, the study acknowledges the complexity of health care systems and provides a structured method to evaluate HIV service integration at the county level [14].

Textbox 1 outlines the framework of the indicator system for the county-level integration of HIV medical and preventive services, based on the Valentijn Rainbow Model. This model highlights the coordination across multiple levels—system, organizational, professional, and clinical—and categorizes indicators into primary dimensions such as policy support, health care accessibility, and service coordination. These indicators are crucial for evaluating the effectiveness of integrated HIV services in rural, county-level regions.

Textbox 1. **Framework of the indicator system for county-level integration of HIV medical and preventive services.**

**System integration**

- Policy support
  - Inclusion of HIV prevention and treatment in county development plans in the past 3 years [15]
  - Formulation of HIV prevention and treatment implementation plans in the past 3 years [16]
- Work system
  - Development of an annual work plan for HIV prevention and treatment in the past year [17]
  - Establishment of incentive measures for HIV prevention and treatment in the past year

**Organizational integration**

- Organizational structure
  - Participation of relevant government departments in HIV prevention and treatment [18]
  - Participation of public health institutions in HIV prevention and treatment
  - Functional complementarity and cooperation among different institutions in HIV prevention and treatment [19]
- Organizational collaboration
  - Signing of agreements between health care institutions for HIV prevention and treatment [17]

**Professional integration**

- Collaboration forms
  - Involvement of senior public health professionals in grassroots HIV prevention and treatment
  - Regular guidance by senior physicians on grassroots HIV prevention and treatment
  - Reasonable professional composition of family physician teams for HIV services
- Coordination mechanism
  - Establishment of chronic disease joint outpatient clinics within the county-level medical consortium [20]
  - Establishment of a communication mechanism for HIV prevention and treatment services among health care personnel [20]
  - Distribution of condoms and other pre-exposure prevention measures in the county

**Clinical integration**

- Accessibility
  - Coverage rate of integrated HIV prevention and treatment services in primary health care institutions
  - Number of newly enrolled people living with HIV receiving treatment
  - Baseline viral-load detection rate [21]
  - CD4 T-cell detection rate [22]
  - Proportion of people with CD4 count < 100 cells/µL [23]
  - Antiretroviral therapy coverage rate [24]
  - Sulfamethoxazole usage rate
- Comprehensiveness
  - Timeliness of infectious-disease reporting
  - Screening rate of high-risk populations for HIV [25]
  - Standardized management rate of people living with HIV [25]
  - Incorporation of traditional Chinese medicine services into integrated HIV prevention and treatment
  - Incidence of HIV-related complications [26]
- Continuity
  - Follow-up rate of people living with HIV [27]
  - Patient loss rate within the year or month
  - Proportion of patients receiving care within 3 months after diagnosis [28]
- Coordination
  - Tracking rate of referred patients [25]
  - Follow-up rate of patients referred back [25]
- Safety
  - Regular viral-load testing [29]
  - Mortality rate within the year or month [29]
- Satisfaction
  - Patient satisfaction [30]
  - Staff satisfaction with integrated HIV services [30]

**Normative integration**

- Funding support
  - Establishment of dedicated funds for integrated HIV prevention and treatment by county governments
  - Proportion of medical-consortium surplus funds allocated to HIV prevention and treatment performance incentives
- Talent support
  - Number of general practitioners per 10,000 population [27]
  - Number of family physician teams per 10,000 population
  - Number of infectious-disease specialists per 10,000 population in secondary hospitals [27]
  - Annual number of special training sessions on integrated HIV prevention and treatment in the county [27]
  - Establishment of an information-system platform for management of patients living with HIV in the county [31]
- Information system
  - Information sharing among health care institutions within the county
  - Utilization rate of electronic health records [29]
  - Coverage rate of telemedicine services in primary health care institutions

**Functional integration**

- Service supervision
  - Supervision and management of integrated medical and preventive services by health administrative departments [17]
  - Development of service procedures for integrated medical and preventive care by health care institutions [32]
- Organizational commitment
  - Participation of health care personnel in integrated medical and preventive services [32]

To refine the framework, FGDs were conducted, engaging 3 public health professors, 10 HIV health care professionals, and 7 graduate researchers. Participants engaged in 3 rounds of discussion, with graduate students systematically reviewing PubMed, Web of Science, China National Knowledge Infrastructure (CNKI), and Wanfang to gather evidence on HIV policies and intervention [33]. The discussion process facilitated an evidence-driven and consensus-based refinement of the tertiary indicators, ensuring that the evaluation framework is both empirically grounded and contextually relevant. The resulting tertiary indicator system is detailed in Textbox 1.

The application of FGDs enhances the practical relevance and stakeholder alignment of the indicator system [33,34]. By incorporating experts with diverse backgrounds, this study ensures that the framework reflects real-world health care challenges and policy priorities. The rationality of the theoretical approach lies in its ability to bridge theory and practice, using the Rainbow Model structured framework to guide integration while refining the indicators through expert consensus. This approach enhances the validity, feasibility, and applicability of the proposed indicator system, making it a valuable tool for evaluating and improving HIV service integration at the county level.

### Delphi Method

The Delphi method, developed by the RAND Corporation [35] in the 1950s, is a structured communication technique that gathers and refines expert opinions through multiple rounds of questionnaires, aiming to achieve consensus on complex issues [36]. In public health, this method is instrumental in areas where empirical evidence is scarce or inconclusive, facilitating the development of guidelines and evaluation frameworks [37,38]. For instance, it has been used to define criteria for selecting peer educators in HIV management in China, ensuring that the chosen individuals are well suited to current practices.

In the context of HIV prevention and treatment in rural China, the Delphi method has been used to develop performance evaluation systems for the CDC at various administrative levels [39]. By engaging experts in iterative consultations, this approach ensures that the resulting evaluation indicators are both contextually relevant and scientifically robust, addressing the unique challenges faced in underdeveloped regions [40].

### Patient and Public Involvement Strategy

Participants engaged in 3 rounds of discussion, with graduate students systematically reviewing PubMed, Web of Science, CNKI, and Wanfang to gather evidence on HIV/AIDS policies and intervention strategies, including those related to HIV service delivery during the COVID-19 pandemic, with a focus on key populations in diverse settings. The search results were systematically analyzed, and experts with a high volume of publications in high-impact journals and a research focus aligned with the study objectives were selected as consulting experts. In addition, we included professionals with more than 5 to 10 years of experience from the Health Commission and CDC [41], as well as medical personnel involved in AIDS prevention and treatment (Textbox 1). Two rounds of consultation questionnaires were then distributed via email. Upon receipt of the feedback, comprehensive data analysis and consistency tests were performed to validate the responses.

### Designing the Delphi Questionnaire

The Delphi questionnaire is designed to develop a 3-tier indicator system for HIV prevention and treatment in China’s counties and rural areas. Its purpose is to achieve consensus on key themes and indicators and to assess the degree of consistency across various focus areas.

### Recruitment Strategy

Recruitment for this study will involve sending emails and letters to 100 targeted individuals. Additionally, recruitment efforts will be extended through networks across China, patient-oriented research support groups, and academic institutions via word-of-mouth and strategic email campaigns. The primary participants will include staff from health and wellness committees, disease control centers, university professors and researchers, and medical personnel involved in AIDS prevention and control.

The use of financial incentives (500 RMB [US $30]) in this study is intended to motivate expert participation and compensate for their time and effort, especially given the multiple rounds of feedback required by the Delphi method. This approach helps ensure a high response rate and minimizes participant attrition. However, there is a potential risk of bias, as experts may feel subtly influenced by the incentive. To mitigate this, it is important to emphasize that participation is voluntary, with a clear assurance that responses will remain anonymous and independent.

To mitigate potential respondent loss, the sample size will be expanded to 200 during the first round of recruitment.

### Study Participants

The selection criteria for experts in this study were based on the recommendations proposed in 1975 by Delbecq, Van de Ven, and Gustafson regarding the selection of Delphi research participants. These recommendations include (1) senior management decision-makers who will use the outcomes of the Delphi study, (2) professionals and their support teams, and (3) respondents to the Delphi questionnaires whose opinions are being solicited [42].

Accordingly, we selected 3 key categories of experts in the field of HIV prevention and control: government officials, health care professionals, and academic researchers. Among them, government officials from disease prevention and control agencies were required to encompass all administrative levels, including the Chinese Center for Disease Control and Prevention (China CDC), provincial CDCs, municipal CDCs, and county-level CDCs. Health care professionals were drawn from tertiary hospitals, township health centers, and community health service centers, ensuring representation across different tiers of the health care system. Efforts were made to achieve a balanced distribution of experts across these various levels [40].

To enhance the representativeness and authority of the expert panel, we established specific inclusion criteria tailored to each category of experts. Determining the appropriate sample size in Delphi studies is crucial for achieving a reliable consensus. While there is no universally prescribed number, research suggests that a sample size of 10 to 15 participants may be sufficient if the expert panel is homogeneous [43]. However, for multistakeholder Delphi surveys, a minimum of 60 to 80 participants is recommended to ensure high replicability of results. Considering the scope and diversity of our study, we aimed to include 60 to 75 experts, balancing feasibility with the need for diverse and comprehensive input [44].

This structured and deliberate approach to expert selection is designed to ensure that the Delphi process yields robust, credible, and actionable insights for advancing HIV prevention and control efforts (Table 1)

**Table 1.** Expert selection criteria for the Delphi study on HIV prevention and treatment integration.

Source	Inclusion criteria	Participants (range)
Government agencies related to HIV prevention and control	At least 5 years of relevant work experience in health commissions or disease prevention and control centers	20‐25
HIV prevention and treatment institutions (hospitals, primary health care service institutions)	At least 5 years of relevant work experience in HIV prevention and control; includes staff from tertiary hospitals, secondary hospitals, community health centers, and township health center or village health unit personnel	20‐25
Research institutes	At least 5 years of experience in HIV-related research and has published related core journal articles as the first author	20‐25

### Subsequent Validation

Subsequent validation of the evaluation framework will include pilot testing in 2 hospitals in Yunnan Province, involving 5 physicians in each hospital. This small-scale, real-world application will test the framework’s feasibility and allow for the collection of feedback from health care professionals. Presurveys will also be conducted to assess the initial perceptions and readiness of the health care staff. Based on the pilot results and feedback, adjustments will be made before broader implementation. The framework will then be evaluated through field implementation across additional county-level areas to assess its effectiveness, applicability, and impact on improving health care service integration.

### Ethical Considerations

The project was reviewed and approved by the Ethics Committee of Tongji Medical College, Huazhong University of Science and Technology (ID: 21YJA630062). This study strictly adheres to the ethical principles of the Declaration of Helsinki. Full voluntary informed consent was obtained from all participants, with clear disclosure of data collection purposes, scope, and secondary use rights, as well as the right to withdraw without prejudice. Collected data was de-identified and encrypted to protect privacy and the study. Participants were compensated up to 500 RMB (US $30).

### Dissemination

The research findings will be presented at international conferences and published in reputable international academic journals. Additionally, a policy report on the “China County AIDS Prevention and Control Evaluation Effect Indicator System” will be submitted to the relevant stakeholders.

### Delphi Process

**Figure 1.**
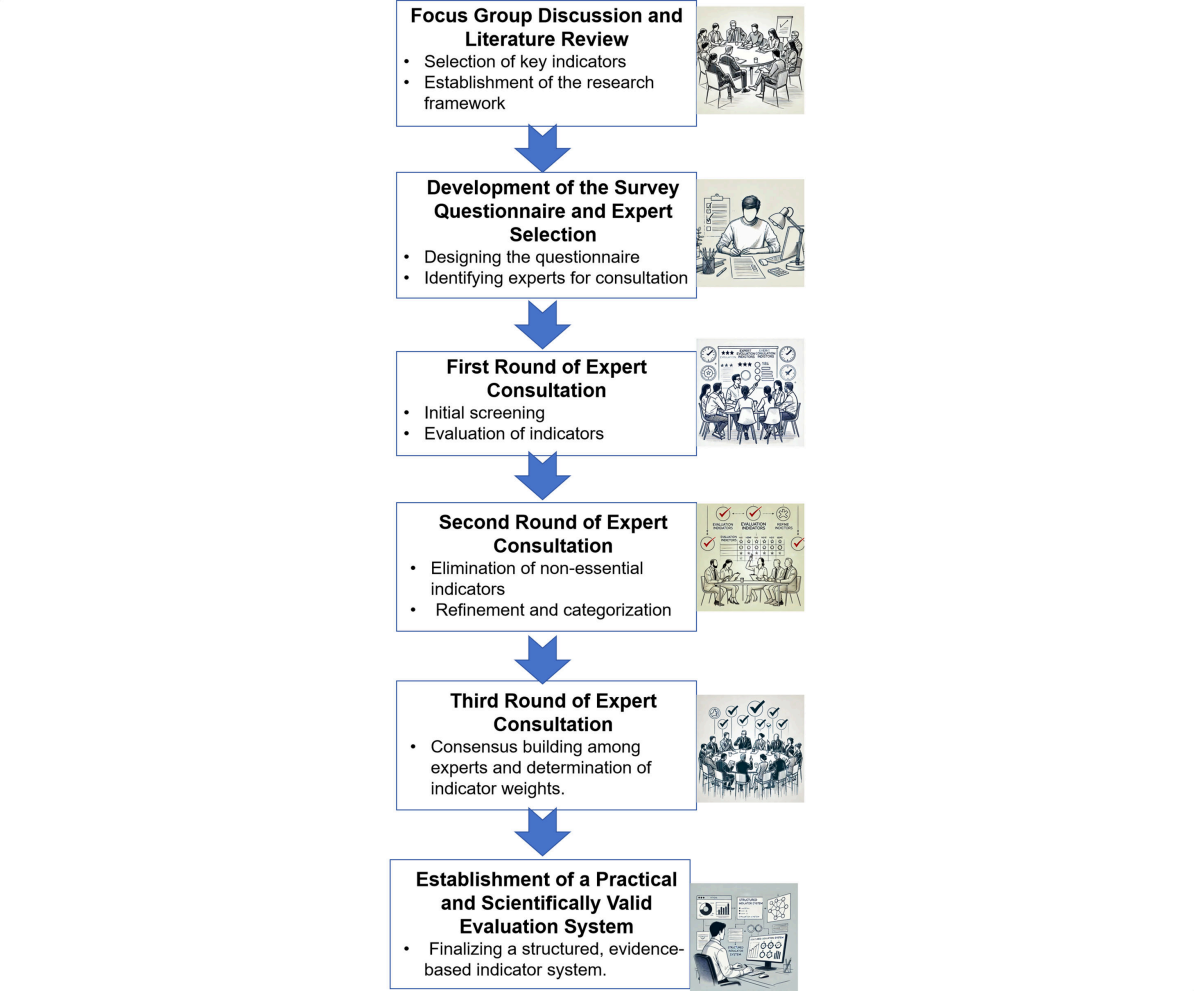
Delphi process.

Based on the WHO guidelines for HIV, we identified key research themes during each round of our Delphi expert consultation. As shown in Figure 1, our process involved 3 rounds of letter-based consultations that ensured active expert participation and refined the indicators until a practical, consensus-based index system was achieved.

### Administration of the Delphi Questionnaire

The study will be conducted from April 1, 2025, to January 31, 2026. The first to third rounds of questionnaires will be distributed using Tencent Documents, from May to September 2025, and qualitative interviews will be conducted using Zoom (Zoom Communications, Inc) and telephone consultation. Respondents who wish to receive a printable version of the questionnaire can choose to receive a PDF version of the questions. The members of the research group will submit a reply through Tencent Documents. Quantitative and qualitative data analysis will be carried out between the 3 rounds of surveys, and the development of the indicator system and the dissemination of the final report will be completed by January 2026.

### First Round

The first round of the Delphi study aims to refine the evaluation framework for the county-level integration of HIV medical and preventive services by eliminating nonessential indicators and dimensions. A panel of 60 to 75 experts is invited to assess the preliminary indicator framework, providing suggestions for additions, deletions, and modifications based on practical experience, theoretical analysis, peer knowledge, and intuitive judgment. Experts rate the importance of each indicator on a 1 to 5 scale, with higher scores indicating greater importance. Their feedback is used to calculate engagement, authority, coordination, and consensus levels, guiding the selection of indicators and ensuring iterative refinement.

Statistical analysis is conducted using SAS (SAS Institute Inc) to compute the mean, SD, coefficient of variation, and Kendall W for each indicator, assessing the degree of expert agreement. Nonparametric tests are applied to determine statistical significance (*P*<.05), indicating whether expert opinions are significantly divergent. Additionally, R (R Foundation for Statistical Computing) is used for analytic hierarchy process (AHP) to derive indicator weights. The consistency ratio is calculated to validate expert agreement in the AHP process, with CR<0.1 indicating acceptable consistency.

Findings from this round will be used to refine the evaluation framework, ensuring that the selected indicators are scientifically robust and relevant to assessing the integration of medical and preventive services for HIV. Expert feedback and statistical validation contribute to the systematic development of a comprehensive and evidence-based assessment system.

### Second Round

The second round of the study focused on identifying the key themes and indicators prioritized by the participants in the first round. During this round, participants were tasked with selecting a predetermined number of topics across multiple dimensions, including prevention and health education for potential patients, diagnosis and treatment, health intervention for patients, and the interface between prevention and treatment. The specific number of topics selected for each category was determined based on insights from the first round, and a weighting calculation was applied to determine the final selection of topics and indicators in similar categories. Topics and indicators that received the highest participant selection advanced to the next round.

Cronbach α was used to assess the internal consistency of the responses in each round. Additionally, 2 members of the research team analyzed the free-text comments provided in the questionnaires to identify relevant changes in the survey topics. These findings were reported in detail to the advisory group, which evaluated the need to add or modify topics and indicators accordingly.

### Third Round

In the third round of the Delphi expert consultation, efforts were made to ensure that the evaluation index system for regional HIV medical and preventive integration was comprehensive, practical, and refined. The research team conducted a collective review of the primary and secondary topics related to HIV integration at the county level, aiming to minimize bias and capture all key concepts to enhance the scientific rigor and practical applicability of the indicator system.

First, indicators and dimensions that received little attention from experts in the first and second rounds were eliminated. Second, the questions were revised to include clear definitions of medical terms and, where possible, to use simpler language, facilitating the implementation of the indicator system in practical evaluations. Finally, participants were informed that they could complete the round in multiple sessions, save their progress, and would receive a reminder if their questionnaire remained incomplete after 1 week.

The purpose of the third round is to accept suggestions on the modification of the index system already obtained by experts in the first 2 rounds, accept feedback, and resolve the uncertainty of the index system determined by the first 2 rounds of expert review. The research team analyzes the advice provided by 2 master’s and PhD experts in order to inform any final adjustments if necessary. These changes will be reviewed and finalized by the research team of 7 master and doctoral students and 2 associate professors. The task force will use these findings to develop a countywide AIDS prevention and control indicator system that best matches actual needs.

## Results

The Delphi study was initiated in April 2025, with 3 rounds of questionnaires distributed between May and September 2025. Data analysis is ongoing, and the finalized county-level HIV integration index system is expected to be published by spring 2026. As of the most recent round, the expert panel has refined the core indicators, with high consensus achieved on key dimensions of service integration. Initial data suggest promising trends in both service accessibility and coordination across county-level health institutions, indicating potential for meaningful improvements in HIV care integration.

## Discussion

### Anticipated Findings

This study aims to systematically develop an evaluation framework for county-level HIV service integration, contributing to both theoretical and methodological advancements in the field. While previous research has explored various metrics for assessing HIV-related health outcomes [45], diagnosis, and treatment effectiveness, no prior study has provided a comprehensive, empirical framework specifically for evaluating the integration of HIV prevention and medical care at the regional level [46]. The key contributions of this study are as follows.

First, this study uses the Rainbow Model as the theoretical foundation to construct an integrated health care service framework, developing a 3D indicator system encompassing medical services, public health interventions, and social support [47]. This model emphasizes a multilayered integration of prevention and treatment, facilitating a more comprehensive assessment of HIV service effectiveness at the country level. Second, the study engages a diverse group of stakeholders, including policymakers, researchers, and health care professionals, ensuring that the evaluation framework incorporates multidisciplinary perspectives and remains relevant to real-world implementation [48]. Finally, this study uses a mixed-methods approach by integrating FGD, the Delphi method, and AHP**,** which helped reduce participant attrition while enhancing the interpretability and practical applicability of the findings.

### Limitations

Despite its contributions, this study has several limitations. First, the developed framework is designed to assess the overall effectiveness of integrated HIV service delivery at a regional level rather than evaluating the impact of specific health education or intervention programs. Therefore, its primary utility lies in policy and system-level evaluations rather than in assessing individual interventions. Second, the literature review is conducted primarily using Chinese-language databases, and the recruited experts are predominantly from China, which may limit the generalizability of the framework to other geographical contexts. Future research should incorporate international datasets and expert input to improve the global applicability of the framework. Third, although the Delphi method and AHP enhance consensus-building, expert judgments remain inherently subjective, potentially influencing the selection and weighting of indicators. Selection bias in expert recruitment may also limit the comprehensiveness of the identified indicators, which is a known limitation of the Delphi process.

The effectiveness of the framework will be measured through both qualitative and quantitative assessments. During the pilot testing phase, we will gather feedback from health care professionals and administrators regarding the framework’s feasibility, ease of use, and impact on service integration. Additionally, key performance indicators such as patient satisfaction, service coordination, and health care outcomes will be tracked during the field implementation phase. We will use statistical methods, including pre- and postimplementation comparisons, to evaluate the framework’s effectiveness in improving HIV service delivery at the county level. We have now explicitly included this in the methods section for clarity.

### Conclusions

The findings of this study will have significant implications for HIV prevention and treatment policies and implementation science. First, this study refines the conceptual framework of HIV service integration, providing a structured approach to enhancing the continuity of care and comprehensive lifecycle services for people living with HIV [48]. Second, by identifying key indicators, this study develops a robust evaluation framework specifically tailored for resource-limited settings, offering policymakers a practical tool for assessing the effectiveness of integrated HIV interventions. Finally, by engaging 75 leading HIV prevention and treatment experts in China, this study contributes to evidence-informed policymaking and promotes cross-disciplinary collaboration in HIV service delivery.

## Abbreviations

AHP: analytic hierarchy process
ART: antiretroviral therapy
CDC: Centers for Disease Control and Prevention
CNKI: China National Knowledge Infrastructure
FGD: focus group discussion
HRSA: Health Resources and Services Administration
NIH: National Institutes of Health
WHO: World Health Organization
